# Supplementary material for: Noncentral forces mediated between two inclusions in a bath of active Brownian rods
Source: Sci Rep. 2021 Nov 29;11:23100. doi: 10.1038/s41598-021-02295-y (PMC8630027; doi:10.1038/s41598-021-02295-y)
Supplement: Supplementary file 1 — Supplementary Information. [file 41598_2021_2295_MOESM1_ESM.pdf]

Supplementary information:

Noncentral forces mediated between two inclusions in a bath of active Brownian rods

Mahmoud Sebtosheikh[1,*] **and Ali Naji**[2,1,*]

[1]School of Physics, Institute for Research in Fundamental Sciences (IPM), P.O. Box 19395-5531, Tehran, Iran
[2]School of Nano Science, Institute for Research in Fundamental Sciences (IPM), P.O. Box 19395-5531, Tehran, Iran
[*]mahmoud-sebtosheikh@ipm.ir; a.naji@ipm.ir

ABSTRACT

This supplementary information includes further discussions on the spatial distribution of noninteracting active Brownian rods around the inclusions, as considered in Subsection 4.3 of the main text.

1 Distribution of noninteracting active Brownian rods

In the case of noninteracting active rods, steric interactions act only between individual rods and the two inclusions and the steric layering effects due to inter-rod interactions will thus be absent. Figure S1 shows the cumulative surface angular density, $Q(\varphi) = \int_0^\sigma \rho(r,\varphi)\,\mathrm{d}r$, of active rods in a narrow layer, or radial interval $[0,\sigma]$, at the immediate vicinity of the right inclusion as a function of φ, the polar angle that measures the angular position around the center of the right inclusion and relative to the x-axis. The plots in panels a to c show the results for the self-propulsion tilt angle $\theta = 0, \pi/4$ and $\pi/2$, respectively, at fixed $d = 0.25\sigma$. In Fig. S1a ($\theta = 0$), $Q(\varphi)$ vanishes except at two narrow sharp peaks that correspond to the active rods being entirely trapped in the upper and lower wedge-shaped gaps between the inclusions. The two peaks show equal amplitudes; hence, the transverse component of the effective force on the inclusions vanishes, leading to a central (longitudinal) interaction between the inclusions. The said interaction is repulsive due to the enhanced accumulation of rods in the intervening gap between the inclusions. Fig. S1b ($\theta = \pi/4$) displays two peaks of unequal amplitudes and, thus, an up-down asymmetry in the distribution of active rods. The concentration of active rods on the upside is higher than that on the downside, creating a clockwise torque on the inclusion dimer. In Fig. S1c ($\theta = \pi/2$), we find a nearly even concentration of active rods in all values of φ, except approximately within the angular range $[5\pi/6, 7\pi/6]$, where $Q(\varphi)$ is zero. This plot indicates that transversally self-propelling rods cover the surface of the inclusions isotropically, except in the intervening region, whence they are sterically excluded. In this case, the active rods effectively push the inclusions toward one another, causing an effective attraction between them.

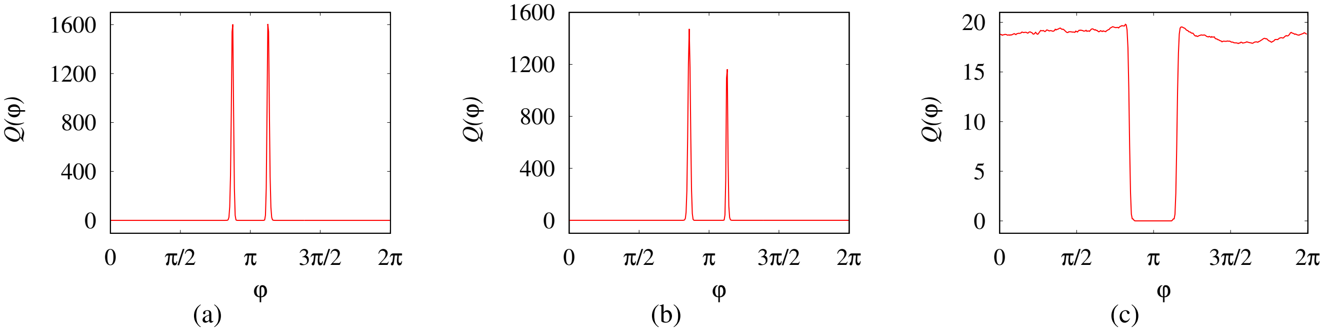

Figure S1. Panels (a), (b) and (c) show the cumulative surface angular density, $Q(\varphi) = \int_0^\sigma \rho(r,\varphi)\,\mathrm{d}r$, of active noninteracting rods as a function of the polar angular coordinate φ around the right inclusion for self-propulsion tilt angles $\theta = 0, \pi/4$ and $\pi/2$, respectively. Here, $\mathrm{Pe} = 20$ and $d/\sigma = 0.25$ are fixed.
